# Potential effects of *Grapevine leafroll-associated virus 3* (genus *Ampelovirus;* family *Closteroviridae*) or *Grapevine red blotch virus* (genus *Grablovirus*; family *Geminiviridae*) infection on foliar phenolic and amino acid levels

**DOI:** 10.1186/s13104-022-06104-1

**Published:** 2022-06-20

**Authors:** Christopher M. Wallis

**Affiliations:** Crop Diseases, Pests and Genetics Research Unit, USDA-ARS San Joaquin Valley Agricultural Sciences Center, 9611 S. Riverbend Ave, Parlier, CA 93648 USA

**Keywords:** Induced defense responses, Phenolics, Amino acids, Plant host resistance, *Vitis vinifera*, *Grapevine leafroll-associated virus 3* (GLRaV-3), *Grapevine red blotch virus* (GRBV)

## Abstract

**Objective:**

Grapevine (*Vitis* spp.) viral infections, including those by *Grapevine leafroll-associated virus 3* (GLRaV-3) and *Grapevine red blotch virus* (GRBV), greatly reduce fruit yields and quality. Evidence exists that host chemistry shifts result in reductions in fruit quality. However, changes over the season in foliar chemistry has not been well examined. Therefore, phenolic and amino acid levels were examined in leaves collected in grapevines with different rootstocks that were healthy or were infected with GLRaV-3 or GRBV. This was part of an effort to assess changes that different pathogens cause in grapevine tissues.

**Results:**

Month and year appeared to account for the greatest variability in grapevine foliar phenolic or amino acid levels, followed by differences in rootstock, and then differences in infection status. GLRaV-3 infection significantly lowered levels of total and individual hydroxycinnamic acid derivatives, and GRBV lowered total phenolic levels, total and individual hydroxycinnamic acids. Amino acid levels were increased over controls in vines infected by GLRaV-3, but not with GRBV. Overall, changes within grapevine leaves due to viral infection were likely too small to overcome variability due to sampling time or rootstock cultivar, and therefore such factors should be considered in determining infection effects on plant foliar chemistry.

## Introduction

Two of the most impactful viral pathogens of grapevine are Grapevine leafroll-associated virus 3 (GLRaV-3) (species *Grapevine leafroll-associated virus 3*; genus *Ampelovirus*; family *Closteroviridae*; order *Martellivirales*) and Grapevine red blotch virus (GRBV) (species *Grapevine red blotch virus;* genus *Grablovirus*; family *Geminiviridae*; order *Geplafuvirales*) [1–3], which can reduce yields and fruit quality necessitating vineyard replacement. Both are currently managed through a combination of vector control (for GLRaV-3), rouging/replanting (for both), and use of certified materials (for both) [4]. However, preventing spread overall remains very challenging and increases in the incidence of these pathogens in vineyards is unavoidable [4].

Despite hypotheses that changes in host physiology due to viral infections would result in observed symptoms, just a few efforts have been made to measure host physiology shifts in afflicted vines [1]. GLRaV-3 was shown to reduce leaf photosynthesis post-veraison [1]. In berries, GLRaV-3 reduced free amino acid levels [5], altered genes involved in phenolic compound and sugar metabolism [6], and possessed lower anthocyanidin content [1, 7, 8]. In leaves, infection resulted in a two- to ten-fold increase in phenolic (flavonoid) production gene expression, and anthocyanidin production occurred resulting in red pigmented leaves [9, 10].

In contrast to GLRaV-3, far less is known about how GRBV effects host chemistry. Most work has focused on GRBV effects on berry quality [11–13]. As for foliage, phenolic compounds increased late in the season in infected than healthy vines, generally in symptomatic tissues [14]. Particular amino acids also were observed as increased in the leaves of infected vines compared to those that were healthy [14].

Most of these studies have observed physiology late in the season, in leaves displaying symptoms [9, 14]. This is the same time of season whereby grapevine viral titers are the greatest and easily detectable by PCR [15, 16].

In contrast to many of these previous studies, this study had an objective to examine whether or not shifts in two major metabolic groups, amino acids and phenolics, could occur prior to symptom expression in GLRaV-3 or GRBV infected grapevines. This was done in asymptomatic tissues, with sampling conducted in May, July, and early September for 2 years (2018 and 2019). For each virus, different rootstock cultivars were utilized to observe rootstock effects on observations. Findings should reveal changes in host biochemistry that in occur in the foliage of grapevines afflicted with GLRaV-3 and GRBV prior to infections, with implications on our understanding of host-viral interactions in asymptomatic plants.

## Main text

### Methods

In the middle of May, July, and September of 2018 and 2019, leaves were collected from healthy or previously infected GLRaV-3 or GRBV grapevines planted at two experimental vineyards established in Davis, CA, USA, and were maintained to commercial standards. These vineyards, one to test GLRaV-3 and another to test GRBV, were established as a larger effort by Foundation Plant Services, University of California, Davis, CA, to examine the impact of viral infections on grape berry yields and quality over time. As such, only a limited number of vines were available for this particular pilot experiment to examine host chemistry. Regarding the GLRaV-3 vineyard, ‘Cabernet Franc’ (*Vitis vinifera*) vines were grafted on either ‘101-14 MGt’ (*Vitis riparia* x. *Vitis rupestris*), ‘Freedom’ (*Vitis riparia* x. *Vitis rupestris* x. *Vitis champinii*), or ‘St. George’ (*Vitis rupestris*) rootstocks, planted in September 2010, and kept healthy or infected by GLRaV-3 since December 2012 (with status verified by PCR in June 2017 and June 2018). Regarding the GRBV vineyard, ‘Cabernet Sauvignon’ (*Vitis vinifera*) vines were grafted on either ‘101-14 MGt’ or ‘St. George’ rootstock, planted in August 2014, and kept healthy or infected by GRBV since September 2016 (with status verified by PCR in February 2018). All infections were made via grafting. Rootstocks and infections were made in a completely, randomized design.

Each vine had three to five fully expanded and mature leaves, approximately the fifth leaf from the apical end, collected from different randomly-chosen branches of the same vine. Leaves were placed into labeled 50 mL centrifuge test tubes, and then immediately flash-frozen in liquid nitrogen. Samples were kept in a − 20 °C freezer until processed for chemical analyses.

Phenolics and amino acids were analyzed using methods from Wallis et al. [17] and Wallis and Chen [18] and using reagents and solvents provided by Thermo-Fisher Scientific (Waltham, MA, USA) unless stated otherwise. Frozen leaf samples were pulverized with a mortar and pestle in liquid nitrogen and had two 0.10 g aliquots weighed out into two 1.5 mL centrifuge tubes. For one of these tubes, the pulverized tissue was twice-extracted overnight at 4 °C in 0.5 mL methanol (for a total of 1 mL of methanol extract). The other tube was twice-extracted overnight at 4 °C in phosphate buffered saline solution (pH 6.8), for a total of 1 mL PBS extract.

High-performance liquid chromatography was used to examine phenolic compounds isolated in methanol. A Shimadzu (Columbia, MD, USA) A LC-20AD pump based liquid chromatograph equipped with Supelco Ascentis RP-18 (Sigma-Aldrich, St. Louis, MO, USA) column and a Shimadzu PDA-20 photodiode array detector had a total of 50 µL of the methanol extract injected into it for each sample. Sigma-Aldrich provided reference compounds used to identify and quantify compounds if available, with additional compounds identified via liquid chromatography-mass spectrometry using a Shimadzu LCMS2020 system [17] and comparing molecular weight information and relative retention times with those previously reported for grapevine stems and roots. Concentrations of the phenolics were made by running standard curves using compounds from the same compound class, such as caftaric acid for hydroxycinnamic acid derivatives or quercetin glucoside for flavonoids.

Amino acids were quantified by using a commercial kit (EZ:FAAST for GC-FID) obtained by Phenomenex (Torrance, CA, USA). For each sample, 100 µL of the PBS buffer extraction was used. Quantification was made using a Shimadzu GC2010 gas chromatography equipped with a flame ionization detector and using the kit-provided column and run method. Kit-provided external and internal standards were utilized to convert peak areas into measurable units.

Statistics were performed using IBM (Armonk, NY, USA) SPSS statistics version 24, with α = 0.05. Normality for all statistical tests was verified by examining deviations of residual plots and the use of Levene’s tests a priori. Summed totals of phenolics, the two major subclasses of phenolics (hydroxycinnamic acid derivatives and flavonoids), and amino acids were analyzed via univariate analyses of variance (ANOVAs) with a general linear model, with year, month, rootstock cultivar, infection status, and all interactions as treatment factors. Prior to these analyses, repeated-measures ANOVAs with the same general model were performed and suggested no significant effects (*P* < 0.05) of repeated sampling on the results. Each virus was handed separately, that is, one analysis was performed for GLRaV-3 and one for GRBV for each compound grouping. When appropriate, follow-up multiple comparison post-hoc Least Significant Difference tests were performed.

### Results and discussion

#### GLRaV-3 effects on grapevine foliar chemistry

**Fig. 1:**
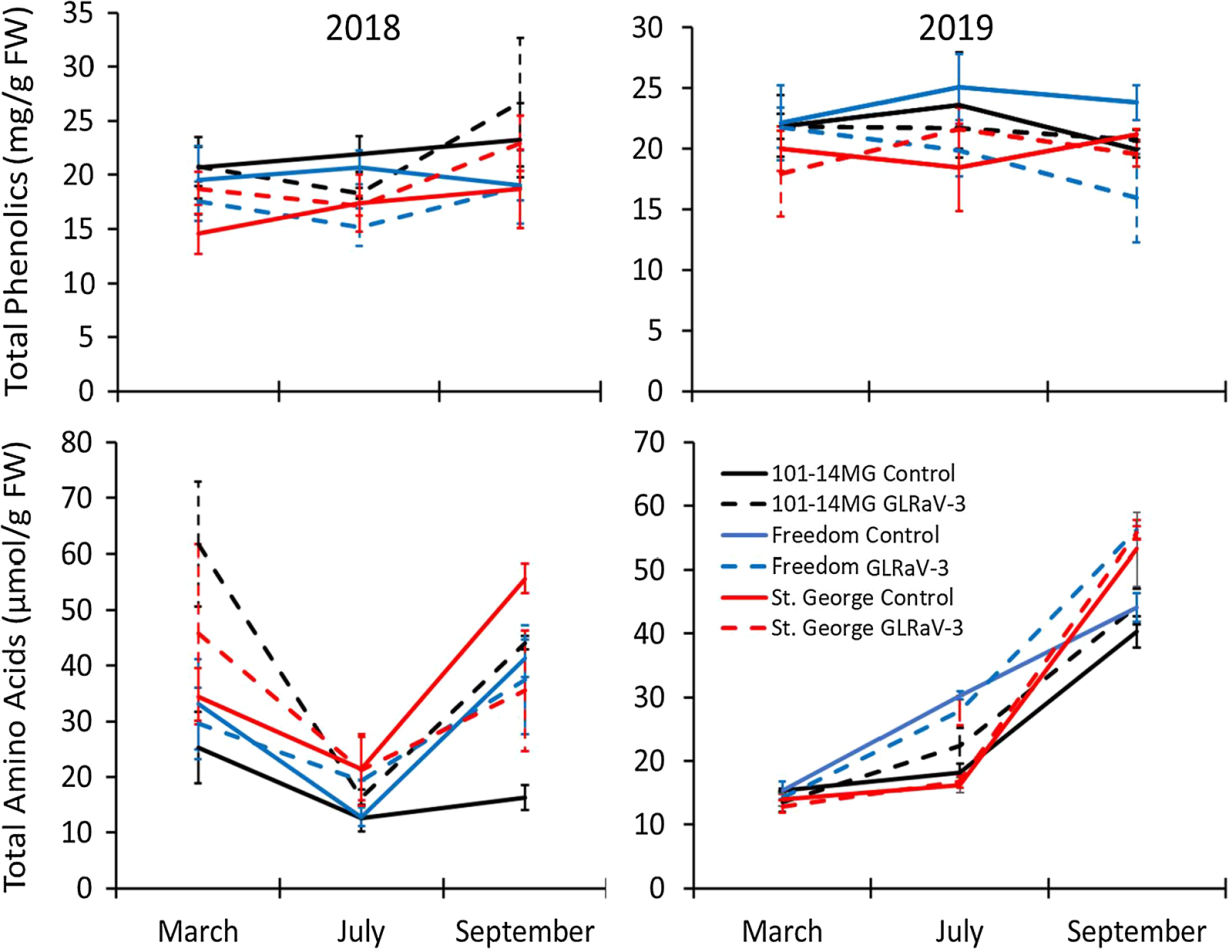
Mean (± SE) total foliar phenolic levels (top panels) or total amino acids levels (bottom panels) in vines grown on one of three different rootstocks and were either healthy or infected by GLRaV-3. Plants were sampled in May, July, or September in 2018 (left panels; N = 53 for either compound types) or 2019 (right panels; N = 53 for amino acids and N = 54 for phenolics)

A total of 21 phenolics were quantified within grapevine leaves harvested in this study, with 10 being putatively identified as hydroxycinnamic acids derivatives and 11 being putatively identified as flavonoids. Total phenolic levels present within GLRaV-3 infected vines did not significantly differ from controls, nor did year or month have significant effects (Table 1; Fig. 1). Rootstock cultivar did have a significant effect, with vines with ‘St. George’ as the rootstock having lower phenolic levels than the other cultivars (Table 1). Total flavonoid levels were only significantly affected by month harvested (greater in September), rootstock (greater in ‘101-14 MG’ vines), and the year by month interaction. Total hydroxycinnamic acid levels were significantly affected by year (greater in 2019), month (greater in July), the year by month interaction, the month by rootstock interaction, and infection status (greater in healthy than GLRaV-3 infected vines) (Table 1).

**Table 1:** Summary of ANOVA and MANOVA results for effects on different grapevine compound classes

Virus	Compound	Factor	F	P	Effects
GLRaV-3	Total phenolics	Infection status	0.936	0.337	
		Month	0.631	0.535	
		Year	2.828	0.097	
		Rootstock	3.772	0.029	101-14MGt > Freedom, St. George
	Total flavonoids	Infection status	0.091	0.764	
		Month	6.439	0.003	Sept > May, July
		Year	0.008	0.928	
		Rootstock	3.918	0.024	101-14MGt > Freedom, St. George
		Year × month	4.756	0.012	
	Total hydroxycinnamic acids	Infection status	6.367	0.014	Healthy > Infected
		Month	33.399	< 0.001	July > May, Sept
		Year	39.693	< 0.001	2019 > 2018
		Rootstock	1.442	0.243	
		Year × month	29.894	< 0.001	
		Month × rootstock	2.719	0.039	
	Total amino acids	Infection status	5.25	0.025	Infected > Control
		Month	62.178	< 0.001	Sept > May > July
		Year	2.63	0.109	
		Rootstock	1.95	0.15	
		Year × month	34.302	< 0.001	
		Month × rootstock	3.398	0.013	
		Rootstock × infection	5.243	0.008	
		Year × rootstock × infection	4.85	0.011	
GRBV	Total phenolics	Infection status	4.218	0.045	Healthy > Infected
		Month	8.76	0.001	July, Sept. > May
		Year	32.917	< 0.001	2019 > 2018
		Rootstock	14.879	< 0.001	101-14MGt > St. George
		Year × month	5.357	0.008	
	Total flavonoids	Infection status	2.313	0.135	
		Month	6.19	0.004	July, Sept. > May
		Year	22.915	< 0.001	2019 > 2018
		Rootstock	15.798	< 0.001	101-14MGt > St. George
		Year × month × rootstock	3.372	0.043	
	Total hydroxycinnamic acids	Infection status	5.08	0.029	Healthy > Infected
		Month	30.044	< 0.001	July > May, Sept
		Year	25.568	< 0.001	2019 > 2018
		Rootstock	2.704	0.104	
		Year × month	37.249	< 0.001	
	Total amino acids	Infection status	0.757	0.389	
		Month	10.008	< 0.001	Sept. > May, July
		Year	2.636	0.111	
		Rootstock	0.096	0.758	
		Year × month	8.787	0.001	
		Month × rootstock	10.274	< 0.001	

Interaction statistics are provided only for interactions that were significant (*P* < 0.05). For GLRaV-3, N = 106 or 107. For GRBV, N = 72

A total of 15 amino acids were quantified in this study. For the GLRaV-3 cohort, significant effects on amino acids levels were observed for month (greater amounts in September), infection status (greater amounts in GLRaV-3 infected vines), year by month interaction, month by rootstock interaction, rootstock by infection status interaction, and year by host by infection status interaction (Table 1; Fig. 1).

It was unexpected to observe greater phenolic levels but fewer amino acids levels in non-infected plants than those afflicted with GLRaV-3. Yet, amino acids such as phenylalanine are precursors to phenolics, so it would be consistent to expect differential effects based on such phenomenon [19]. Greater amino acid levels also could imply infected leaves were a greater nutrient sink than leaves from uninfected plants.

#### GRBV effects on grapevine foliar chemistry

**Fig. 2:**
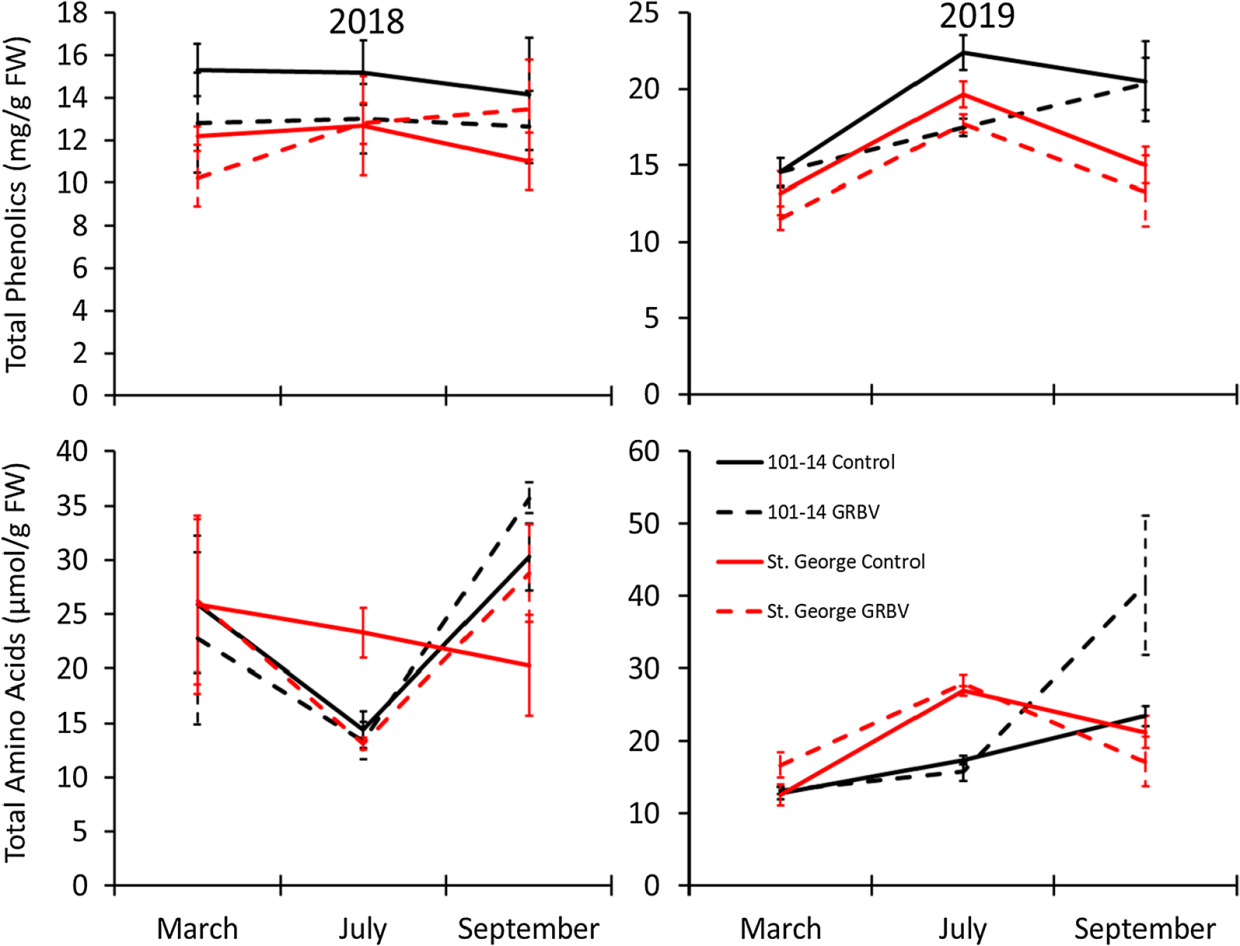
Mean (± SE) total foliar phenolic levels (top panels) or total amino acids levels (bottom panels) in vines grown on one of two different rootstocks and were either healthy or infected by GRBV. Plants were sampled in May, July, or September in 2018 (left panels; N = 36 for either compound type) or 2019 (right panels; N = 36 for either compound type)

For the GRBV cohort, total phenolic levels differed due to year (greater in 2019), month (greater in July), rootstock cultivar (greater in ‘101-14 MGt’ vines), infection status (greater in controls than GRBV infected vines), and year by month interaction (Table 1; Fig. 2). Total flavonoid levels were significantly affected by year (greater in 2019), month harvested (greater in July), and rootstock cultivar (greater in ‘101-14 MGt’ vines) (Table 1). Total hydroxycinnamic acid levels were significantly affected by year (greater in 2019), month (greater in July), the year by month interaction, and infection status (greater in healthy than GRBV infected vines) (Table 1).

For the GRBV cohort, significant effects on amino acids levels were observed for month (greater amounts in September), year by month interaction, and month by rootstock interaction (Table 1; Fig. 2).

Similarly to infection by GLRaV-3, infection by GRBV has significantly reduced foliar phenolic levels. Once again, this perhaps suggests mild viral infections keep leaves as sinks longer and delay differentiation and development.

### Conclusions

Overall, phenolic and amino acid levels were much more affected by sampling time or rootstock cultivar than infection status. This suggests that weather, phenology, and influences from the rootstock utilized drove phenolic or amino acids levels more than infection status. This would compromise efforts to utilize host physiology-based diagnostic techniques, such as those that might look at subtle changes in leaf color to find viral infected plants prior to fully symptomatic development [20].

Lastly, unlike previous studies [9, 14], this study aimed to collect non-symptomatic foliage to observe potential changes in host chemistry over a greater timeframe throughout the growing season. Clearly this had an effect on findings, and also demonstrated limitations of analyzing host chemistry in mature, field-grown vines experiencing climatic fluctuations throughout the year. Great care should be taken in future studies to control variability to reach more accurate conclusions about viral infection effects on grapevine metabolites such as amino acids and phenolics.

### Limitations

These findings were limited by lack of replication which should be improved in similar future efforts. Furthermore, a greater number of both cultivars and locations should have been considered, as these were observed to be major drivers of variability within this study. One solution for future studies could be collecting a larger pool of leaf samples representing many more vines throughout the vineyard, thus reducing potential micro-environmental effects. Lastly, conclusions from this experiment were limited by lack of viral titer assessments. However, in the collected tissues titers were likely too low to be detected by PCR due to known issues with PCR-based detections of these viruses during certain times of the growing season [15, 16].

## Data Availability

The data described in this Research Note can be freely and openly accessed on the USDA Ag Data Commons (https://doi.org/10.15482/USDA.ADC/1524456).

## Abbreviations

ANOVA: Analysis of variance
GLRaV-3: *Grapevine leafroll-associated virus 3*
GRBV: *Grapevine red blotch virus*
